# 

**Published:** 1892-01

**Authors:** 


The second annual session of the Association of Military Surgeons of the National Guard of the United States will be held at St. Louis, April 19, 20, and 21, 1892. An interesting programme of addresses by prominent surgeons of the National Guard and the United States Army has been arranged, and a goodly number of scientific papers on Military and Accidental Surgery will be read and discussed, and all matters pertaining to the health, usefulness and welfare of the civilian soldiers will receive attention. Dr. Nicholas Senn, of Chicago, is President; Dr. Frederick L. Matthews, Springfield, Ill., is Secretary, and Dr. Eustathius Chancellor, of St. Louis, is Chairman of the Committee of Arrangements.

### BOOKS AND PAMPHLETS RECEIVED.

International Clinics: A Quarterly of Clinical Lectures on Medicine, Surgery, Gynecology, Pediatrics, Neurology, Dermatology, Laryngology, Ophthalmology, and Otology. By Professors and Lecturers in the leading Medical Colleges of the United States, Great Britain, and Canada. Edited by John M. Keating, M. D., Philadelphia, Consulting Physician for the Diseases of Women to St. Agnes' Hospital, Philadelphia; Editor Cyclopedia of the Diseases of Children.—J. P. Crozer Griffith, M. D., Philadelphia, Clinical Professor of Diseases of Children in the University of Pennsylvania; Professor of Clinical Medicine in the Philadelphia Polyclinic.—J. Mitchell Bruce, M. D., F. R. C. P., London, England, Physician and Lecturer on Therapeutics at the Charing Cross Hospital, and David W. Finlay, M.D., F.R.C.P., London, England, Physician to the Middlesex Hospital, and to the Royal Hospital for the Diseases of the Chest; Lecturer on Clinical Medicine in the Middlesex Hospital Medical School. July, 1891. Philadelphia: J. B. Lippincott Company. 1891.

Transactions of the Association of American Physicians. Sixth session, held at Washington, D. C., September 22, 23, 24, and 25, 1891. Volume VI. Philadelphia: Printed for the Association. 1891.

The Physician as a Business Man; or, How to Obtain the Best Practical Results in the Practice of Medicine. By J. J. Taylor, M. D. Philadelphia: *The Medical World*, 1520 Chesnut St. 1891.

A Manual of Hypodermatic Medication: The Treatment of Diseases by the Hypodermic or Subcutaneous Method. By Roberts Bartholow, A. M., M. D., LL. D., Emeritus Professor of Materia Medica, General Therapeutics and Hygiene in the Jefferson College of Philadelphia; Honorary Fellow of the Royal Society of Edinburgh; Honorary Member of the Société Medico-Practique de Paris; Fellow of the College of Physicians of Philadelphia; Member of the American Philosophical Society; Fellow of the Medico-Chirurgical Faculty of Maryland; Honorary Member of the various States and local Societies; Author of a Treatise on the Practice of Medicine; of a Treatise on Materia Medica and Therapeutics; of a Manual of Medical Electricity, etc., etc. Fifth edition, revised and enlarged. Philadelphia: J. B. Lippincott Company. London: 10 Henrietta street, Covent Garden. 1891.

History of the Circumcision from the Earliest Time to the Present. Moral and Physical Reasons for its Performance, with a History of Eunichism, Hermaphrodism, etc., and of the Different Operations Practiced upon the Prepuce. By P. C. Remondino, M. D. (Jefferson), Member of the American Medical Association; of the American Public Health Association; of the San Diego County Medical Society; of the State Board of Health of California, and of the Board of Health of the City of San Diego; Vice-President of the California Medical Society, and of the Southern California Medical Society, etc. Philadelphia and London: F. A. Davis. 1891.

Saunders' Pocket Medical Formulary, with an Appendix, containing Posological Table, Formulæ and Doses for Hypodermic Medication; Poisons and their Antidotes; Diameters of the Female Pelvis and Fetal Head; Diet List for Various Diseases; Obstetrical Table; Materials and Drugs used in Antiseptic Surgery, etc. By William M. Powell, M. D., author of Essentials of Diseases of Children; one of the Associate Editors of the Annual of the Universal Medical Sciences ; Attending Physician to the Children's Seashore House for Invalid Children, and the Mercer House for Invalid Women. at Atlantic City, N. J.; Member of the Philadelphia Pathological Society. Formerly Instructor of Physical Diagnosis in University of Pennsylvania ; Attending Physician to the Children's Clinic at the University of St. Clement's Hospital ; Chief of the Medical Clinic of the Philadelphia Polyclinic. Philadelphia : W. B. Saunders, 913 Walnut street. 1891.

Transactions of the Ophthalmological Section of the American Medical Association at the Forty-second Meeting, held at Washington, D. C., May 5 to 8, 1891.

A Treatise on Practical Anatomy for Students of Anatomy and Surgery. By Henry C. Boenning, M. D., Lecturer on Anatomy and Surgery in the Philadelphia School of Anatomy ; Demonstrator of Anatomy in the Medico-Chirurgical College ; Demonstrator of Anatomy in the Philadelphia Dental College ; Lecturer on Disease of the Rectum in the Medico-Chirurgical College, etc. Illustrated with 198 wood engravings. Philadelphia and London : F. A. Davis, Publisher. 1891.

Manual of Physical Diagnosis, for the Use of Students and Physicians. By James Tyson, M. D., Professor of Clinical Medicine in the University of Pennsylvania, and Physician to the University Hospital ; Fellow of the College of Physicians of Philadelphia ; Member of the Association of American Physicians, etc. Philadelphia : P. Blakiston, Son & Co., 1012 Walnut street. 1891.

A Manual of Venereal Diseases : Being an Epitome of the Most Approved Treatment. By Everett M. Culver, A. M., M. D., Pathologist and Assistant Surgeon Manhattan Hospital of New York City ; Member of the American Association of Andrology and Syphilology, and late of the Department of Venereal Diseases of the Vanderbilt Clinic ; and James R. Hayden, M. D., Chief of Clinic Venereal Department of Vanderbilt Clinic, College of Physicians and Surgeons, New York. With illustrations. Philadelphia : Lea Brothers & Co. 1891.

A Practical Treatise on the Diseases of the Ear, including a Sketch of Aural Anatomy and Physiology. By D. B. St. John Roosa, M. D., LL. D., Professor of the Diseases of the Eye and Ear in the New York Post-Graduate Medical School, and President of the Faculty ; Surgeon to the Manhattan Eye and Ear Hospital ; Consulting Surgeon to the Brooklyn Eye and Ear Hospital ; formerly Professor of Ophthalmology in the University of the City of New York, and of Diseases of the Eye and Ear in the University of Vermont ; formerly President of the Medical Society of the State of New York, etc., etc. Seventh revised edition. New York : William Wood & Co. 1891.

Age of the Domestic Animals. Being a Complete Treatise on the Dentition of the Horse, Ox, Sheep, Hog, and Dog, and on the Various Means of Determining the Age of these Animals. By Rush Shippen Huidekoper, M. D., Veterinarian (Alford, France) ; Professor of Sanitary Medicine and Veterinary Jurisprudence American Veterinary College, New York ; Lieutenant-Colonel and Surgeon-in-Chief National Guard of Pennsylvania ; Fellow of the College of Physicians, Philadelphia ; Honorary Fellow Royal College of Veterinary Surgeons, London ; late Dean of the Veterinary Department, University of Pennsylvania, etc. Illustrated with 200 engravings. Philadelphia and London : F. A. Davis, Publisher. 1891.

A B C of the Swedish System of Educational Gymnastics. A Practical Hand-Book for School Teachers and the Home. By Hartvig Nissen, Instructor of Physical Training in the Public Schools of Boston, Mass, ; Instructor of Swedish and German Gymnastics at Harvard University's Summer School, 1891 ; formerly Instructor of Physical Culture at the Catholic University, Washington, D. C., etc., etc. With seventy-seven illustrations. Philadelphia and London : F. A. Davis, Publisher. 1891.

The Supreme Passions of Man; or, The Origin, Causes, and the Tendencies of the Passions of the Flesh. Setting Forth the Results of Scientific Inquiries into the Appetites of Mankind, and the Passions which they Excite. A Study of the Crimes of the Flesh, and the Efforts of Christianity to Maintain Purity. An Essay on the True Causes of Drunkenness, and the Only Way to Prevent this Evil. Observations on the Relation of Vice to the Laws of Nations, and Existing Educational Systems. By Paul Paquin, M. D., late Professor of Comparative Medicine, and Director of the Bacteriological Laboratory, Missouri State University ; Member of the American Public Health Association ; Member of the American Medical Association ; Member of the American Society of Microscopists ; Member of the Missouri State Medical Association, etc., etc., Editor of the *Bacteriological World ;* Director of the Laboratory of Hygiene, Battle Creek, Mich. Published by the Little Blue Book Co., Battle Creek, Mich.

Therapeutics of Diphtheria. By Joseph Burghardt, Practising Physician in Vienna. From a lecture delivered at the meeting of the Council of Vienna Physicians, April 8, 1889. Translated by Charles Raettig. Translation authorized by the lecturer. New York : J. H. Vail & Co. 1891.

A Vegetable Plate ; also a New Technique in Intestinal Anastomosis. By Robert H. M. Dawbarn, M. D., Professor of Surgical Anatomy and Operative Surgery New York Polyclinic.

Some Clinical Features of Stone in the Bladder. By L. Bolton Bangs, M. D., Surgeon to St. Luke's and Charity Hospitals ; Professor of Genito-Urinary and Venereal Diseases in the New York Post-Graduate Medical School. Reprint : *The New York Medical Journal*, August 15, 1891.

The Third Series of One Hundred Consecutive Operations, Showing the Results of Antiseptic Methods of Work. By Robert T. Morris, M. D., New York City, Instructor of Surgery at the Post-Graduate Medical School, New York City. Reprint : *New England Medical Monthly.* Danbury, Conn.: The Danbury Medical Printing Co. 1891.

An Attempt to Show What New Jersey Surgeons Have Done in Abdominal Surgery. By Edward J. Ill, M. D., of Newark, N. J., Fellow of the American Association of Obstetricians and Gynecologists ; Surgeon to Woman's Hospital ; Gynecologist to St. Barnebas' Hospital ; Consulting Surgeon to the German Hospital, etc. Read before the Medical Society of the State of New Jersey. Newark, N. J.: L. J. Hardham, printer and binder, 243 and 245 Market street.

Is a Child Viable at Six and a Half Months ? By Llewllyn Eliot, M. D., Washington, D. C. Reprint : *The Virginia Medical Monthly,* November, 1891.

